# The first case of deafness‐dystonia syndrome due to compound heterozygous variants in *FITM2*

**DOI:** 10.1002/ccr3.1719

**Published:** 2018-07-26

**Authors:** Aamina Shakir, Alexandrea F. Wadley, Gabriela Purcarin, Klaas J. Wierenga

**Affiliations:** ^1^ University of Oklahoma College of Medicine Oklahoma City Oklahoma; ^2^ Section of Genetics Department of Pediatrics University of Oklahoma Health Sciences Center Oklahoma City Oklahoma; ^3^ Division of Pediatric Neurology Department of Neurology University of Oklahoma Health Sciences Center Oklahoma City Oklahoma

**Keywords:** deafness‐dystonia syndrome, *FITM2*, Siddiqi syndrome

## Abstract

We report the second known family affected by deafness‐dystonia syndrome associated with loss of function of *FITM2*. Our patient is compound heterozygous for pathogenic *FITM2* variants, while affected siblings in the first report were homozygous. This case provides evidence that this novel genetic disorder is associated with autosomal recessive inheritance.

## 1 INTRODUCTION

We report a boy with severe developmental delay, dystonia, upper and lower limb contractures, and progressive bilateral sensorineural deafness. He was found to have biallelic loss‐of‐function variants in *FITM2*, a gene in which loss‐of‐function variants were recently reported to be associated with a novel disorder called Siddiqi or deafness‐dystonia syndrome. To date, this syndrome has only been reported in a single consanguineous Pakistani family. Our patient has the main features (dystonia and deafness) of this syndrome, even though he lacks additional features found in the Pakistani siblings, such as ichthyosis and sensory neuropathy. This is the sixth case, but only the second family with deafness‐dystonia syndrome, and it should help to widen the spectrum of clinical features in this emerging condition, as well as to shed light on previously unreported pathogenic variants in *FITM2*.

“Siddiqi syndrome” is a proposed deafness‐dystonia syndrome with presumed autosomal recessive inheritance first described by Zazo Seco et al^5^ in five of eight Pakistani siblings with consanguineous parents. The affected siblings presented with progressive hearing loss, dystonic limb movements leading to immobility and contractures, failure to thrive, neuropathic pain, and ichthyosis‐like skin findings with scarring alopecia. Furthermore, all were found to have homozygous truncating pathogenic variants in *FITM2* (MIM 612029). These five siblings were the first known cases of a disease phenotype ascribed to loss of *FITM2*. Here, we report the second case: A 4‐year‐old boy with compound heterozygous predicted loss‐of‐function variants in *FITM2*, whose phenotype shows significant overlap with that of the previously reported cases.

## 2 CLINICAL REPORT

The patient is a 4‐year‐old boy who is the first living child born to nonconsanguineous parents with unremarkable family histories. Maternal history is notable for a prior miscarriage at 12 weeks. The patient was born via emergency C‐section at 34 weeks’ gestational age due to decreased fetal heart rate and decreased umbilical cord blood flow noted on ultrasound. At birth, he weighed 2 lbs. 8 oz. (<3rd percentile) and was noted to have bilateral adducted thumbs, bilateral correctable planovalgus foot deformities, and borderline low‐set ears with no other abnormalities. He required a 23‐day NICU stay that was complicated by a grade 1 bilateral intraventricular hemorrhage.

He initially presented to pediatric endocrinology at age of 7 months for failure to thrive, global developmental delay, and subjective hyperactivity. Thyroid panel was within normal limits, including serum total T3 and serum reverse T3, thus ruling out Allan‐Herndon‐Dudley syndrome. He was referred to genetics shortly afterward, where oligonucleotide chromosomal microarray was ordered with negative results. At 10 months, he continued to have delayed milestones, with poor head control and inability to sit unsupported. He also displayed left‐sided preference and axial dystonia. MRI brain showed underdevelopment of the inferior vermis of the cerebellum, consistent with mild Dandy‐Walker deformity. Bilateral hand X‐rays showed delayed bone age with absent ossification centers and abnormal flexion of the left first metacarpal phalangeal joint. Orthopedic surgery consult at 1 year of age confirmed bilateral hypoplastic thumbs, planovalgus deformity of both feet, and dystonic movement of upper and lower extremities. At that point, neurology's working diagnosis was dyskinetic cerebral palsy possibly due to an unidentified genetic syndrome. He was started on gabapentin with acceptable response.

At 2 years of age, he was referred to neurogenetics for continued delay, significant lower extremity hypotonia, and severe head lag. Testing for *L1CAM* variants to rule out MASA (mental retardation, aphasia, shuffling gait, and adducted thumbs) syndrome was negative. Normal results upon testing for H19 hypomethylation and uniparental disomy of chromosome 7 also made Russell‐Silver syndrome less likely. Around the same time, he underwent a sleep study that demonstrated mild obstructive sleep apnea. Subsequent adenotonsillectomy with direct laryngoscopy revealed tracheal collapse consistent with tracheomalacia or hypotonia. Lipid panel was normal.

Whole‐exome sequencing was performed when the patient was 3 years old, and revealed biallelic predicted loss‐of‐function variants in *FITM2*, a maternally inherited c.39dupC, p.T14DfsX138, and a paternally inherited c.652C>T, p.Q218X. At the time, *FITM2* was not known to be associated with any disorder in humans. However, shortly after receiving the report, in February 2017, Zazo Seco et al published a paper proposing a new genetic disorder called Siddiqi syndrome, a deafness‐dystonia syndrome that has thus far only been identified in a single consanguineous Pakistani family in which all affected members are homozygous for a nonsense variant (c.4G>T, p.E2X) in *FITM2*. While our patient had passed his newborn hearing screen and not previously shown any signs of hearing loss, his mother expressed concern that his hearing was deteriorating around the same time. A sedated brainstem auditory–evoked response (BAER) was subsequently performed and demonstrated profound bilateral sensorineural hearing. The patient has recently received bilateral cochlear implants.

## 3 DISCUSSION

Deafness‐dystonia syndromes are a heterogeneous group of disorders, of which only a few have identified gene/phenotype relationships. The deafness‐dystonia syndromes with known genetic causes are frequently associated with defects in energy metabolism. The disorder due to *FITM2* deficiency appears to follow this trend. *FITM2* is a 2‐exon gene whose protein product, FIT2, is required for normal fat storage and metabolism due to its involvement in partitioning triglycerides into lipid droplets. Kadereit et al^3^ first identified FIT2 and its counterpart FIT1 and found them to be located exclusively in the endoplasmic reticulum membrane. FIT2, conserved from *Saccharomyces cerevisiae*, is a 6‐transmembrane domain protein considered the archetype of the FIT (for *fat‐inducing transcript*) protein family^2^. It is thought to function downstream of diglyceride acyltransferase in the triacylglycerol synthesis pathway, where it diverts triglycerides and diacylglyceride from this pathway and binds them directly within micelles, thus facilitating their package into lipid droplets. Lipid droplets are organelles consisting of a hydrophobic triglyceride core, surrounded by a phospholipid monolayer and associated proteins. Their primary function is thought to be triglyceride storage within adipose tissue, but they likely also play a role in skeletal muscle mitochondrial respiration, by releasing fatty acids for oxidation. Of note, FIT2 is expressed most highly in adipose tissue in mice, but in skeletal muscle in humans. Several mouse models support the importance of FIT2 (and, by extension, lipid droplets) in fat storage and metabolism. Miranda et al^4^ found that mice with adipose‐specific FIT2 deficiency developed severe, progressive lipodystrophy with fatty liver, tissue macrophage infiltration, and insulin resistance, with few but abnormally large lipid droplets on histology. Goh et al^1^ found that whole‐body FIT2 knockout mice developed lethal enteropathy with death from severe fat malabsorption occurring within weeks.

*FITM2* variants were not known to produce disease in humans until Zazo Seco et al reported a novel variant of a deafness‐dystonia syndrome due to homozygous loss‐of‐function variants in *FITM2*. All 5 of the affected siblings had progressive sensorineural hearing loss, extremely limited speech, delayed walking and regression of motor function, and ichthyosis‐like skin scaling and alopecia most prominent at the shins. Three complained of burning peripheral neuropathy‐like pains in the extremities, while two complained of joint pains; three experienced dystonic limb movements with resultant contractures. The oldest sibling was the only one to develop seizures, chronic diarrhea, and urinary incontinence in adolescence. Interestingly, however, none of the patients developed lipodystrophy despite the role of FIT2 in lipid partitioning. Liver fat content and serum triglycerides were also normal. A *Drosophila* model was used to support the *FITM2*'s causative role in the patients’ phenotypes as opposed to the possibility of mutations in other genes due to consanguinity. RNAi downregulation of *Fitm* (the *Drosophila* analog of the *FITM* family in humans) produced corresponding findings of deafness and flightlessness. Similar to humans, the Fitm knockdown flies displayed no specific symptoms related to lipid metabolism; however, they were found to have a significantly reduced lipid droplet size.

Our patient displays remarkably similar features to the patients in the family described by Zazo Seco et al. Like them, his symptoms are most clearly ascribable to FIT2 insufficiency, as WES showed otherwise no relevant alterations. Furthermore, while homozygous variants in other genes could provide alternative explanations for some of the symptoms in the consanguineous Pakistani family, the fact that our patient is from a nonconsanguineous family strengthens the evidence that loss of FIT2 is indeed the cause of Siddiqi syndrome. Of note, however, is the fact that our patient seems to have a pure deafness‐dystonia syndrome without the dermatologic and neuropathic findings shared by all the affected Pakistani siblings. It could be that deafness and dystonia are the hallmark features and perhaps the Pakistani siblings’ other features were due to another gene alteration, given their consanguineous background. It could also be that our patient is still very young and has not yet developed all the features of Siddiqi syndrome. He has a history of gastrointestinal problems, with multiple hospital visits for recurrent vomiting and various GI infections; this aspect of his history may represent a syndromic overlap with the oldest sibling in Zazo Seco et al's family, especially given the evidence of enteropathy in mouse models, but it just as well may result from confounding factors. Regardless, this case should help to further define the phenotype for what Zazo Seco et al propose to call “Siddiqi syndrome.” Over time, we may learn more information about this condition and its natural history and more readily understand the role of the *FITM2* gene.

## CONFLICT OF INTEREST

None declared.

## AUTHORSHIP

AS: wrote case report. AW: edited case report. GP: edited case report. KW: edited and helped to write case report.
